# A novel post-transcriptional role for ubiquitin in the differential regulation of MHC class I allotypes^☆^

**DOI:** 10.1016/j.molimm.2012.10.015

**Published:** 2013-09

**Authors:** Florencia Cano, Paul J. Lehner

**Affiliations:** Cambridge Institute for Medical Research, Department of Medicine, University of Cambridge, Cambridge CB2 0XY, UK

**Keywords:** 3′ UTR, Antigen presentation, HLA-A, MHC class I, mRNA decay, NK cells, Ubiquitin E3 ligase, Ubiquitin

## Abstract

By providing ligands for Cytotoxic T-Lymphocytes (CTL) as well as Natural Killer (NK) cells, the HLA-A/B/C MHC class I molecules (MHC-I) play a central role in both innate and adaptive immunity. In addition to CTL-mediated recognition of MHC-peptide complexes, cell surface expression of MHC-I is closely monitored by NK cells, whose killer-cell immunoglobulin-like receptors encode activatory and inhibitory receptors with specificity for MHC-I. How the cell surface expression of MHC-I is tightly controlled is not well understood. In a functional siRNA ubiquitome screen to identify E3 ligases involved in MHC-I regulation we recently found that MEX-3C, a novel RNA-binding ubiquitin E3 ligase, is responsible for the post-transcriptional, HLA-A allotype-specific regulation of MHC-I. MEX-3C expression is increased upon NK cell activation and modulates the threshold of killing by these cells. We find that MEX-3C binds the 3′-untranslated region of *HLA-A2* mRNA, inducing its RING-dependent degradation. The RING domain of MEX-3C is not required for HLA-A2 cell surface downregulation, but regulates the degradation of *HLA-A2* mRNA. We have therefore uncovered a novel post-transcriptional pathway for regulation of HLA-A allotypes and provide a direct link between ubiquitination and mRNA decay.

## Highlights

► MEX-3C, a novel RNA-binding ubiquitin E3 ligase, regulates *HLA-A* mRNA stability. ► MEX-3C therefore regulates HLA-A, but not HLA–B/C cell surface expression. ► MEX-3C specifically regulates *HLA-A*, but not *HLA–B*/*C*, and modulates NK cell killing. ► MEX-3C binds the 3′UTR of *HLA-A* mRNA, inducing its RING-dependent degradation. ► MEX-3C provides a direct link between ubiquitination and *MHC-I* mRNA decay.

## 1 Introduction

The Major Histocompatibility Complex class I (MHC-I) antigen presentation pathway plays a well-recognized role in defence against intracellular pathogens and tumors; as well as in the establishment of tolerance, autoimmunity and neuronal function (Wearsch and Cresswell, 2008; Shatz, 2009). Almost all nucleated cells express MHC-I molecules at the cell surface where they present antigenic peptides for surveillance by cytotoxic T lymphocytes (CTLs). The CTL-mediated recognition of foreign peptides leads to killing of the infected cells. Cell surface expression of MHC-I is also closely monitored by Natural Killer (NK) cells where signals received by their killer-cell immunoglobulin-like receptors (KIRs) balance the NK-cell response between tolerance of healthy cells and killing of unhealthy ones.

Given the importance of MHC-I in the modulation of both innate and adaptive immune responses, its cell surface expression must be finely tuned. MHC-I molecules are closely regulated at each stage of the antigen-presentation pathway, including transcription, assembly of the complex in the ER, trafficking through the secretory pathway and regulated turnover at the plasma membrane (Wearsch and Cresswell, 2008).

The classical MHC-I multigene family is encoded by three loci, HLA-A, -B, and -C. How the diversity of HLA-A, -B, and -C molecules contribute to their function is incompletely understood. HLA-A and -B are well expressed on most cells and account for the majority of defined CTL epitopes, while cell surface expression of HLA-C is lower, and appears to be the dominant MHC-I in NK cell regulation (Blais et al., 2011). The HLA-A and -B loci have evolved separately with HLA-B evolving more rapidly than either HLA-A or -C. The principal source of HLA-B diversity arises from intralocus recombination, with less gene recombination but more point mutations seen in the HLA-A locus (Parham, 2005). HLA-B alleles play a dominant role in the control of human pathogens, particularly well described in the case of HIV whose control is influenced by the CD8 T-cell response (Kiepiela et al., 2004). Selective loss of expression of alleles of the HLA-A locus (Kageshita et al., 1993; Ferrone and Marincola, 1995), particularly HLA-A2, the commonest HLA allele in Caucasians, is a common feature of tumors and may facilitate CTL escape.

## 2 Current status

Much effort has focused on the transcriptional regulation of MHC-I genes, with particular emphasis on interferon (IFN) or cytokine-mediated upregulation which may be upregulated upon infection (van den Elsen et al., 2004; Johnson, 2003). Type I and II IFNs may also increase MHC-I by promoting mRNA stabilization, as demonstrated in DCs, and the IFN-γ mediated post-transcriptional regulation of individual MHC-I alleles is reported for HLA-B7 and HLA-A2 (Kuchtey et al., 2005). In the case of HLA-A2, this IFN-γ mediated upregulation is due to an increase in its mRNA nuclear export (Browne et al., 2006).

The discovery of large numbers of non-coding RNAs and their interaction with RNA-binding proteins (RBPs) to regulate mRNA levels in key cellular processes has renewed interest in RNA metabolism. Regulation of *MHC-I* mRNA decay was first described almost two decades ago for HLA-C (McCutcheon et al., 1995), and more recently HLA-Cw*0702 mRNA was shown to be post-transcriptionally regulated through the binding of miR-148 (Kulkarni et al., 2011).

Although best recognized for its role in post-translational protein regulation, an intriguing role for ubiquitin in the regulation of RNA stability has emerged from the observations that several ubiquitin E3 ligases encode RNA binding domains (RBDs) and are therefore predicted to bind and regulate RNA (Cano et al., 2010). Indeed, 15 RING-containing E3 ligases possess one or more RBDs. We recently performed a functional siRNA ubiquitome screen which identified MEX-3C as a novel RNA-binding E3 ubiquitin ligase responsible for the post-transcriptional regulation of all common HLA-A allotypes, without affecting the expression of HLA-B and -C. These data suggest a novel mechanism for MHC-I allotype-specific regulation, and for the first time provide a direct link between ubiquitination and *MHC-I* mRNA decay (Cano et al., 2012).

## 3 MEX-3C mediated regulation of *HLA-A* mRNA stability in innate immunity

MEX-3C is a conserved RNA-binding ubiquitin E3 ligase involved in mRNA decay, and belongs to a family of four mammalian proteins (MEX-3A–D), homologues to *Caernorhabditis elegans* MEX-3 (Buchet-Poyau et al., 2007). Each member contains two tandem repeat RNA-binding KH domains and a ubiquitin E3 ligase RING domain. The founder protein, MEX-3 in *C. elegans* contains two KH domains which bind to the 3′UTR of its target mRNA *PAL-1* and inhibits its translation (Hunter and Kenyon, 1996; Draper et al., 1996). Evolutionary diversification from *C. elegans* to *Drosophila* led to the acquisition of a C-terminal ubiquitin E3 ligase RING domain. Two further consecutive rounds of duplication from a single gene in *Drosophila* (dMex-3: RNA-binding + RING finger domain) accounts for the presence of four genes on different chromosomes in higher eukaryotes, including humans (hMEX-3A–D). Therefore, the critical question is why an RNA-binding protein involved in RNA regulation acquired E3 ligase activity? We showed that the presence of the RING was not required for translational repression of HLA-A. However, acquisition of the RING domain allows MEX-3C not only to repress HLA-A translation, but also induce *HLA-A* mRNA degradation. Hence, MEX-3 gene products were initially RNA-binding and later acquired ubiquitin E3 ligase activity, which not only prevents mRNA translation, but also promotes its degradation in a RING-dependent manner (Cano et al., 2012).

MEX-3C is highly expressed in cells of the innate immune system, in particular NK cells. Its expression increases upon NK cell activation as will occur when these cells enter an inflammatory environment. Increased MEX-3C expression will lead to a decrease in cell surface HLA-A levels and alter the threshold of killing by these cells. MEX-3C binds the 3′UTR of the *HLA-A* target mRNA through its RNA-binding KH domains, though the precise sequence specificity and affinity of MEX-3C has proved difficult to define and probably relates to the complex secondary structure of this region (Cano et al., 2012). MEX-3C shuttles with its mRNA cargo from the nucleus to the cytosol via the CRM-1 nuclear export pathway where it interacts with the Argonaute proteins AGO1 and AGO2, and is likely recruited to the RISC-mediated mRNA decay pathway (Buchet-Poyau et al., 2007). How and where the mRNA is sequestered in the absence of ubiquitin ligase activity, and which proteins are ubiquitinated to promote mRNA degradation, remains to be determined. The simplest model would suggest that after trafficking from the nucleus to the cytosol, and subsequent release from its USP7 DUB regulator (Cano et al., 2012), MEX-3C is autoubiquitinated and degraded, allowing the specific mRNA substrate to be transferred to the RNA degradation machinery. mRNAs are protected from decay by their 5′-CAP and the 3′ poly(A) tail. To initiate mRNA decay requires exposure of the mRNA ends, through decapping or deadenylation, to 5′-to-3′ or 3′-to-5′ exonucleases (Mühlemann and Lykke-Andersen, 2010). This disassembly of mRNPs is essential to allow access of the degradation machinery to the targeted mRNA. An alternative function of MEX-3C might be to ubiquitinate components of the mRNP complex, promoting disassembly of the complex and therefore allowing access to the exonucleases.

## 4 Future perspectives

**Fig. 1:**
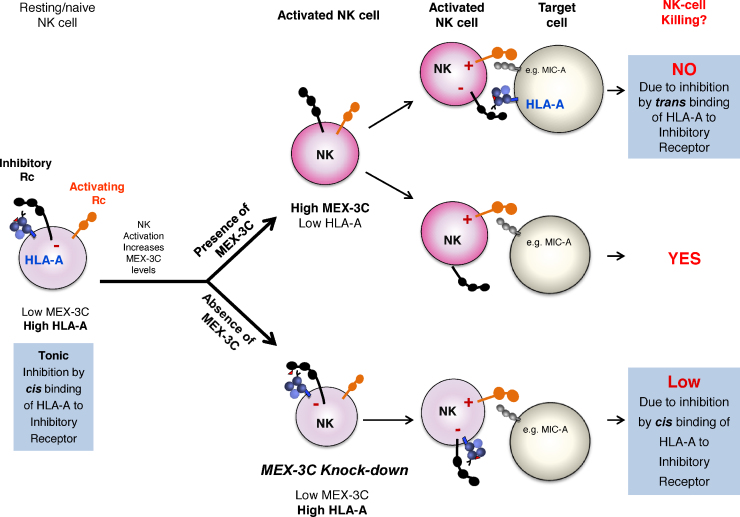
MEX-3C specifically regulates HLA-A in NK cells and modulates NK cell killing. Cell surface expression of MHC-I is closely monitored by NK cells, whose activity is regulated by the balance between positive signals from activating receptors and negative signals from MHC-I recognizing inhibitory receptors. NK Inhibitory receptors can bind MHC-I in *trans* (between opposing membranes, i.e.: on the target cell), as well as in *cis* (on the same cell membrane). In naive/resting NK cells, inhibitory receptors constitutively bind MHC-I in *cis*, causing a tonic suppression that prevents spontaneous cellular activation. NK cell activation increases MEX-3C levels, which results in a decrease in surface expression of HLA-A, freeing the potential inhibitory receptor from *cis* binding, and therefore decreasing the threshold for NK-mediated killing. In this scenario, if the activated NK cell encounters a “healthy cell”, the MHC-I on the target cell engages with the inhibitory receptor in *trans* resulting in no NK-mediated killing of the target cell. However, if the target cell has low expression of MHC-I, then the NK inhibitory receptor is not engaged. This relieves NK cell inhibition, causing the killing of the target cell. In the absence of MEX-3C, however, HLA-A levels on the surface of the NK cell remain high and bound in *cis* to the inhibitory receptor, resulting in a decreased level of NK-mediated killing.

The discovery of MEX-3C and HLA-A allotype-specific regulation raises several questions. For example, what is the requirement and relevance of HLA allotype-specific regulation in the immune system, in particular NK cells? Why NK cells decrease cell surface HLA-A expression upon activation is not known, though the most plausible explanation is to reset the threshold of inhibitory receptors (e.g. KIRs, LILRs) for NK cell killing. Inhibitory receptors can bind MHC-I in *cis* (on the same cell membrane) as well as in *trans* (between opposing membranes, i.e.: on the target cell) (Doucey et al., 2004; Masuda et al., 2007). Such interactions were initially reported between murine Ly49 and MHC-I where *cis* H2D^d^ sequesters Ly49A, relieving NK cells from the inhibitory effect of unengaged Ly49A (Held and Mariuzza, 2008; Chalifour et al., 2009). Conversely, human LILRB2 (and its mouse orthologue PIRB) constitutively bind MHC-I in *cis*, causing a tonic suppression that prevents spontaneous cellular activation (Masuda et al., 2007). The crystal structure of the LILRB1 and HLA-A2 complex revealed that LILRB1 binds HLA-A2 in *trans* as well as in *cis* (Willcox et al., 2003). In this context, the increased levels of MEX-3C seen upon NK cell activation will cause a decrease in cell surface expression of HLA-A, freeing the potential inhibitory receptor from *cis* binding, and therefore decreasing the threshold for NK-mediated killing (Fig. 1). This explanation is consistent with our observations that depletion of MEX-3C increases HLA-A expression and results in decreased NK cell killing (Cano et al., 2012). By downregulating only the HLA-A allele, activated NK cells might escape fratricide as they still express other MHC-I alleles at the cell surface. It also provides a plausible explanation for a resting tonic inhibition on NK cells, which would be unleashed as they become activated. Alternatively, MEX-3C might be required for the resolution of the inflammatory response. The downregulation of HLA-A by MEX-3C following NK activation would render NK cells susceptible to killing by neighbouring NK cells. Such a scenario does not explain the allotype-specific effect, nor the decrease in HLA-A occurring early, rather than late, during resolution of the response (Cano et al., 2012). Finally, low HLA-A expression may also protect NK cells against increased killing from CTLs at sites of inflammation.

It is likely that MEX-3C binds and regulates additional mRNA species. The identification of new mRNA targets will provide further insight into the function and physiological role of this novel E3 ligase, and possibly the whole family of RNA-binding E3 ligases. Expression of MEX-3C in other cell types will likely regulate a variety of cellular processes.

The identification and requirement for MHC-I allotype-specific regulation in the immune system was unanticipated and suggests either novel functions of MHC-I molecules, or differences between HLA-A and -B molecules which have not been appreciated. For example, as noted above many of the HLA associations with the control of infections are related to the HLA-B alleles, as seen in HIV, with few related to HLA-A alleles. In contrast, allotype-specific downregulation is seen in some tumors, such as malignant melanomas which selectively downregulate HLA-A2, -A28 alleles (Kageshita et al., 1993) presumably to facilitate immune evasion. Whether these observations represent major differences between HLA-A and -B alleles, or that these associations are simply non-representative is unclear. It will also be interesting to determine whether a similar level of selective regulation occurs for the HLA-B/C allotypes. Since there are at least 15 RNA-binding proteins with E3 ubiquitin ligase activity, some of these may be involved in the regulation of other MHC-I alleles. Further studies of these ligases will shed light on this novel level of regulation.

In summary, we have uncovered a novel post-transcriptional mechanism for regulating HLA-A alleles by MEX-3C. This finding links ubiquitination and *MHC-I* mRNA decay, and suggests an unanticipated requirement for the allotype-specific regulation of MHC-I molecules.
